# Supplementary figures and images for: Investigation of chemical structure recognition by encoder–decoder models in learning progress
Source: J Cheminform. 2023 Apr 12;15:45. doi: 10.1186/s13321-023-00713-z (PMC10100163; doi:10.1186/s13321-023-00713-z)

## Slide 1
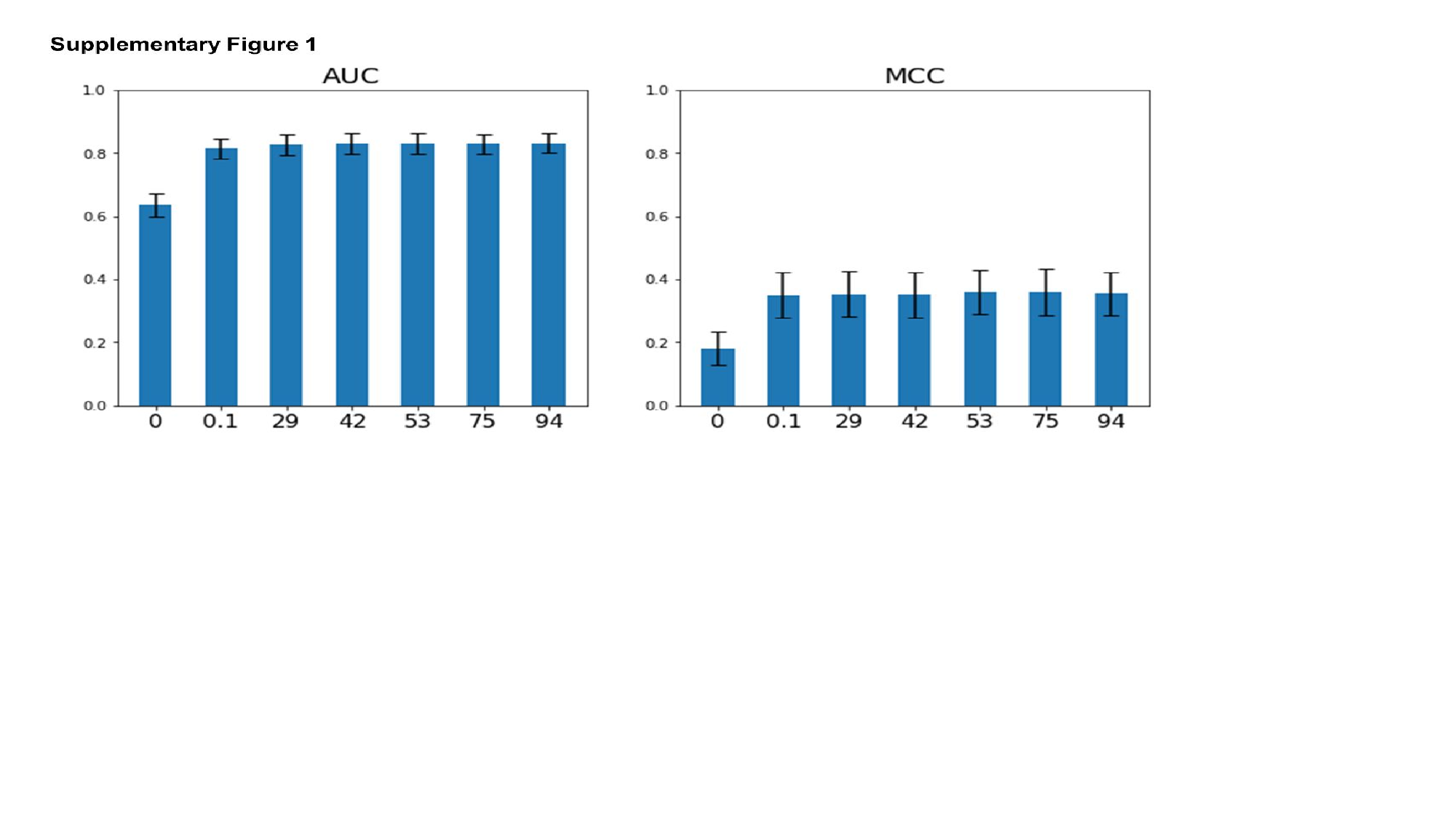

Supplement: Supplementary file 4 — Additional file 4. AUC and MCC of 113 assays prediction compared between perfect accuracy of Encoder–Decoder models. Bar height and error bar indicate mean and standard deviation. [file 13321_2023_713_MOESM4_ESM.pptx]

## Slide 1
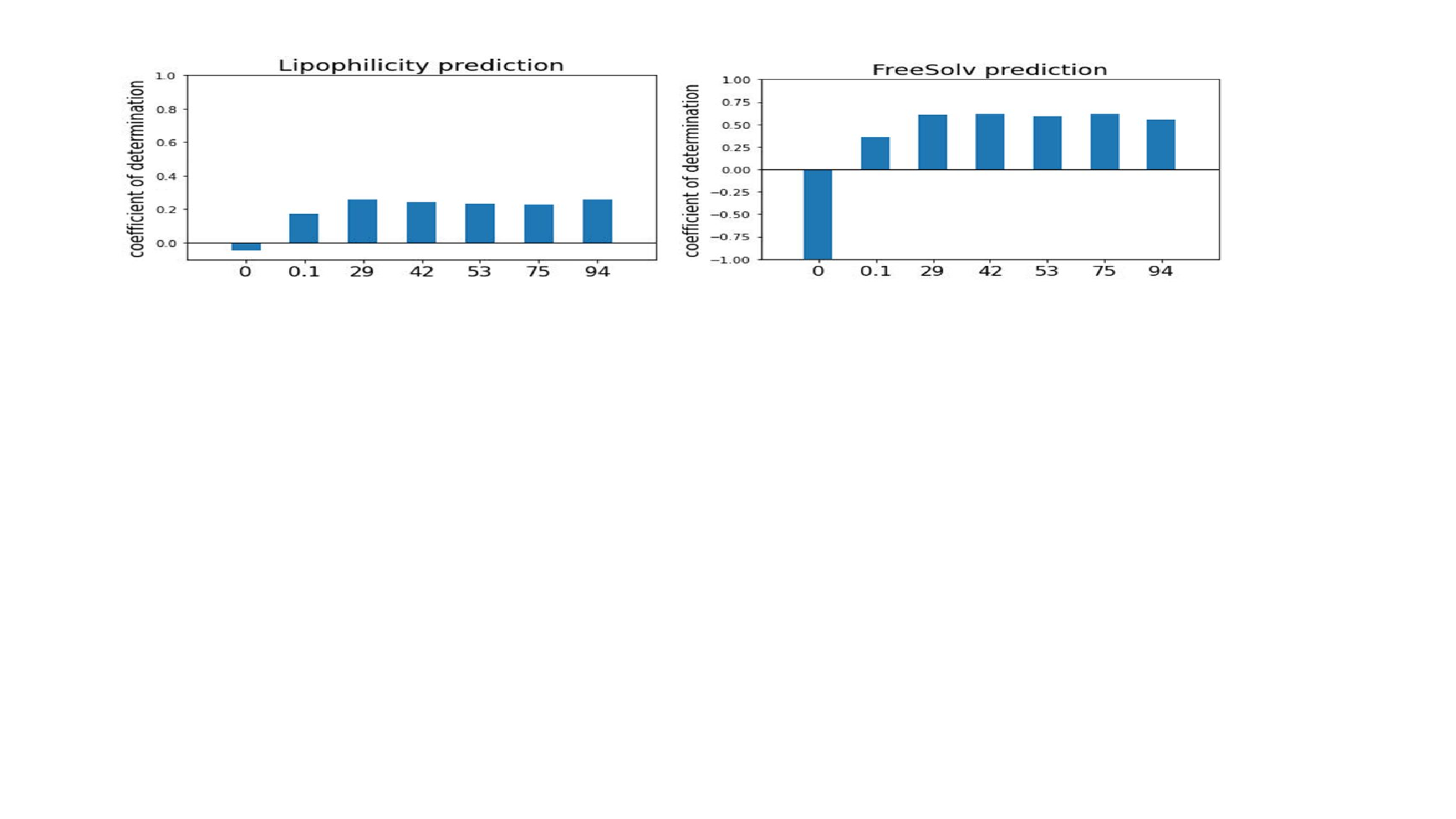

Supplement: Supplementary file 5 — Additional file 5. Coefficient of determination of Lipophilicity and FleeSolv prediction compared between perfect accuracy of Encoder–Decoder models. Lipophilicity and FleeSolv data were obtained from MoleculeNet (https://moleculenet.org/). XGBoost was used as machine learning algorithm for prediction. Hyperparameters listed in Additional file 1 were optimized using Optuna for each dataset prediction with optimization index of RMSE and n_trials of 50. [file 13321_2023_713_MOESM5_ESM.pptx]

## Slide 1
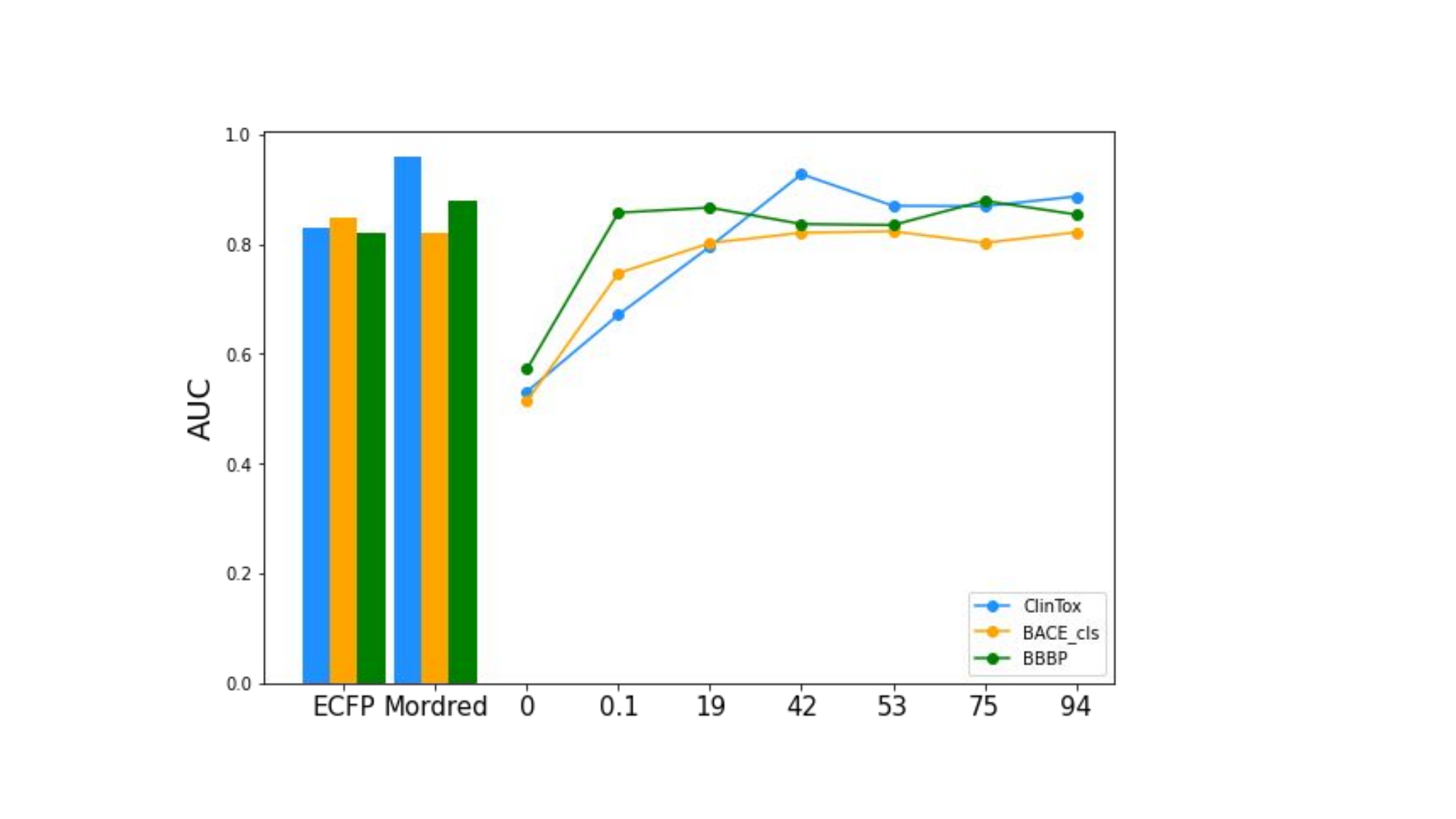

Supplement: Supplementary file 6 — Additional file 6. AUC of ClinTox, BACE, BBBP prediction compared between perfect accuracy of encoder–decoder models, ECFP and Mordred. [file 13321_2023_713_MOESM6_ESM.pptx]

## Slide 1
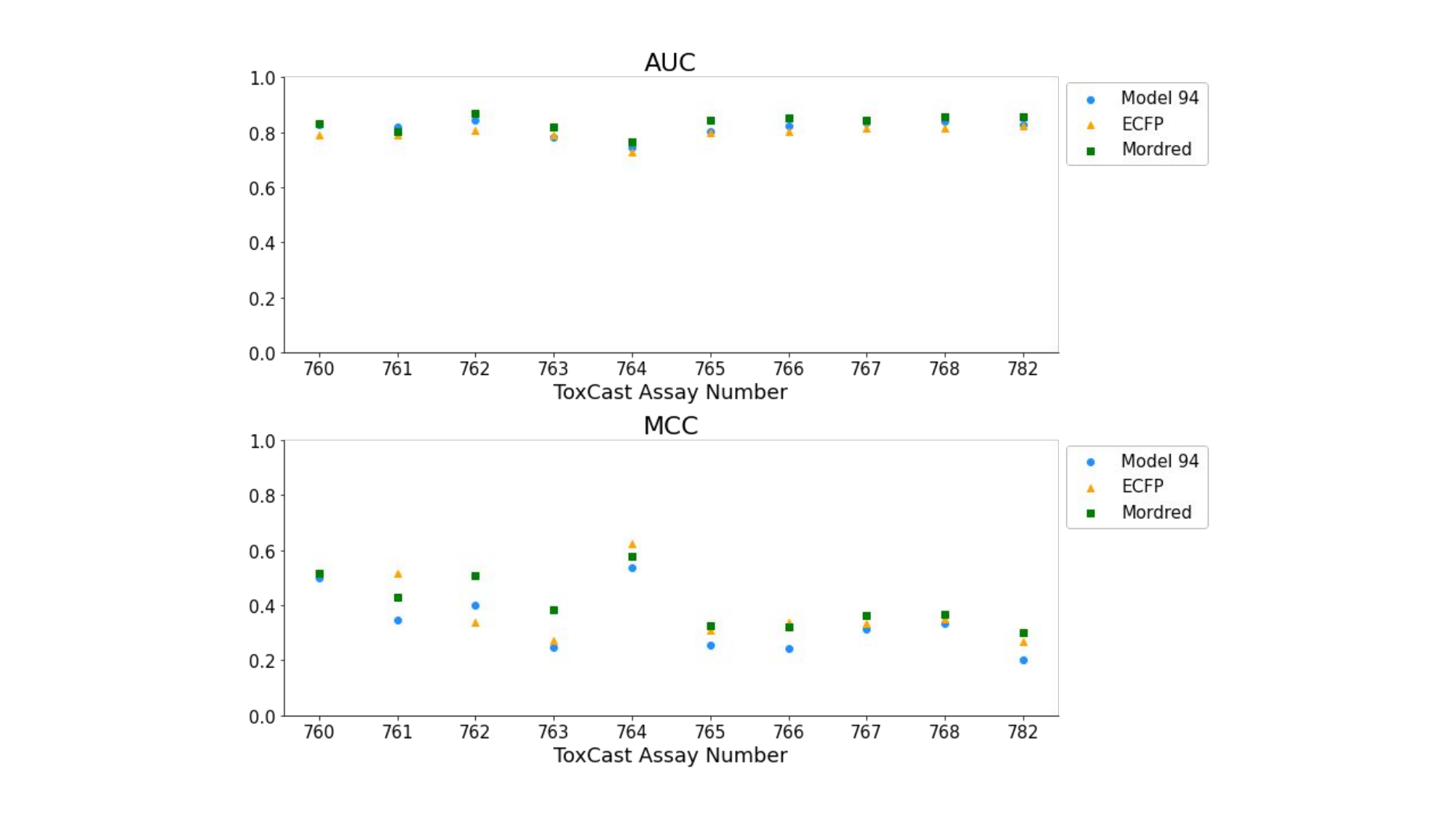

Supplement: Supplementary file 7 — Additional file 7. AUC and MCC of representative 10 assays prediction compared between encoder–decoder model with 94 % perfect accuracy, ECFP and Mordred. [file 13321_2023_713_MOESM7_ESM.pptx]
